# Application of Neural Networks for classification of Patau, Edwards, Down, Turner and Klinefelter Syndrome based on first trimester maternal serum screening data, ultrasonographic findings and patient demographics

**DOI:** 10.1186/s12920-018-0333-2

**Published:** 2018-02-13

**Authors:** Aida Catic, Lejla Gurbeta, Amina Kurtovic-Kozaric, Senad Mehmedbasic, Almir Badnjevic

**Affiliations:** 1 Department of Genetics and Bioengineering, International Burch University, Francuske revolucije bb, Ilidza, 71210 Sarajevo, Bosnia and Herzegovina; 2 Institute for Gynecology, Perinatology and Infertility “Mehmedbasic”, Grbavicka 6a, 71000 Sarajevo, Bosnia and Herzegovina; 3 Department of Clinical Pathology, Cytology and Human Genetics, Clinical Center of the University of Sarajevo, Sarajevo, Bosnia and Herzegovina; 4 Verlab Ltd Sarajevo, Sarajevo, Bosnia and Herzegovina; 5 Faculty of Electrical Engineering, University of Sarajevo, Sarajevo, Bosnia and Herzegovina; 6 Technical Faculty Bihac, University of Bihac, Bihac, Bosnia and Herzegovina

**Keywords:** Combined test, Trisomy, Fetal aneuploidy, Prenatal diagnosis, Artificial neural networks, Feedforward neural network, Feedback neural network

## Abstract

**Background:**

The usage of Artificial Neural Networks (ANNs) for genome-enabled classifications and establishing genome-phenotype correlations have been investigated more extensively over the past few years. The reason for this is that ANNs are good approximates of complex functions, so classification can be performed without the need for explicitly defined input-output model. This engineering tool can be applied for optimization of existing methods for disease/syndrome classification. Cytogenetic and molecular analyses are the most frequent tests used in prenatal diagnostic for the early detection of Turner, Klinefelter, Patau, Edwards and Down syndrome. These procedures can be lengthy, repetitive; and often employ invasive techniques so a robust automated method for classifying and reporting prenatal diagnostics would greatly help the clinicians with their routine work.

**Methods:**

The database consisted of data collected from 2500 pregnant woman that came to the Institute of Gynecology, Infertility and Perinatology “Mehmedbasic” for routine antenatal care between January 2000 and December 2016. During first trimester all women were subject to screening test where values of maternal serum pregnancy-associated plasma protein A (PAPP-A) and free beta human chorionic gonadotropin (β-hCG) were measured. Also, fetal nuchal translucency thickness and the presence or absence of the nasal bone was observed using ultrasound.

**Results:**

The architectures of linear feedforward and feedback neural networks were investigated for various training data distributions and number of neurons in hidden layer. Feedback neural network architecture out performed feedforward neural network architecture in predictive ability for all five aneuploidy prenatal syndrome classes. Feedforward neural network with 15 neurons in hidden layer achieved classification sensitivity of 92.00%. Classification sensitivity of feedback (Elman’s) neural network was 99.00%. Average accuracy of feedforward neural network was 89.6% and for feedback was 98.8%.

**Conclusion:**

The results presented in this paper prove that an expert diagnostic system based on neural networks can be efficiently used for classification of five aneuploidy syndromes, covered with this study, based on first trimester maternal serum screening data, ultrasonographic findings and patient demographics. Developed Expert System proved to be simple, robust, and powerful in properly classifying prenatal aneuploidy syndromes.

## Background

A normal human cell is made up of 46 chromosomes that are grouped into homologous pairs (or classes): 44 autosomes, and two sex chromosomes, which specify gender (XX for female and XY for male) [1]. Each chromosomal homologous pair consists of one maternal and one paternal chromosome that pair up with each other inside a cell during meiosis [2]. Chromosomal disorders fall into two main categories such as numerical and structural abnormalities [3]. Chromosomal anomalies or aneuploidy, represented primarily by numerical change, are the single greatest contributor to prenatal morbidity and mortality [4]. Since chromosomal abnormalities are powerful in detection and diagnosis of various genetic disorders, chromosome analysis (karyotyping) is a fundamental clinical procedure most frequently performed in genetic laboratories. There are several reasons for referral for cytogenetic analysis, but advanced maternal age is still the major reason. Maternal age is a major factor in producing aneuploidy in humans. The most frequent anomaly associated with maternal age is Trisomy 21 [5]. Affected fetuses can be identified early in pregnancy through amniocentesis, thus providing the woman with the option for selective termination or continuation of the pregnancy. It is most important that accurate genetic testing and counseling is provided.

All obstetricians generally offer first trimester maternal serum screening for aneuploidy to their pregnant patients, irrespective of patient’s age. This minimally invasive screening test provides patient with a risk assessment and is not to be used as definitive diagnosing tool. If the results of maternal serum screening are concerning and suggestive of trisomies the patient may opt for confirmatory diagnostic methods, which would require patient to undergo an invasive procedure amniocentesis or chorionic villus sampling (CVS). A diverse range of diagnostic tests are currently available for the detection of prenatal chromosomal aberrations. Karyotyping, fluorescence in situ hybridization (FISH), quantitative fluorescence polymerase chain reaction (QF-PCR), array comparative genomic hybridization (aCGH), and the next-generation sequencing (NGS) are the common methods used for prenatal diagnostics [4–10].

Karyotyping analysis aims to assess the possible presence of genetic defects, identify individual chromosomes in a metaphase cell and arrange them in order based on the established atlas [11]. Today, use of image processing and artificial intelligence techniques has considerably increased in many medical practice fields. In automated cytogenetics, general computerized image processing and analysis techniques as well as rule based classification algorithms for karyotyping have been in use, since the 70s, replacing the human based cutting up chromosome photographs with scissors and their human based arrangements.

Developing tools for disease classification can be extremely extensive and challenging task, especially when the association between input and target values is non-linear and depending on multiple factors [12]. Machine learning methods such as Artificial Neural Networks (ANNs) have been considered as promising tools for overcoming these difficulties since they do not require analytical model of observed process [12]. The theory of neural networks is still growing field due to their ability to derive meaning from complicated or imprecise data and because they use different approach, parallel data processing instead of algorithmic approach to problem solving like conventional computers. Different ANN architectures have been used for various purposes, such as classification, pattern recognition, prediction, control and optimization [13]. The neural networks, in terms of data processing, mimic physical structure of human nervous system consisting of artificial neurons. These units serve as processors that are interconnected and organized into layers [14]. The relationship between input and output is determined by the network architecture and learning algorithm [14]. Learning is an iterative process of adjusting ANN inner parameters, weights and biases until the performance criteria is met. Most usually that performance criteria is threshold of error function. The ability that differentiates ANNS to other data processing tools is the ability to learn and improve its performance from examples. Once trained, ANN is able to predict unknown future outcomes of the same process. ANNs can be classified into two groups based on internal information flow: feedforward and feedback neural networks. Most commonly used type of feedforward architecture is the one with back propagation learning algorithm and as for feedback neural network, Elman’s architecture is most commonly used.

The usage of ANN in disease classification happens very often [15–27], though there have been only few studies investigating neural networks in genome-enabled predictions and classifications [28, 29]. As for cytogenetic analysis, in recent years, several research groups have developed and tested different ANNs for the classification of metaphase chromosomes. The main goal in these studies was to develop automated computer-assisted banded chromosome detection, using various methods and various ANNs architectures for classification of all 24 types of chromosomes [30–34]. Although many efforts have been made to develop computerized schemes for automated karyotyping and syndrome diagnosis, no schemes can get be performed without substantial human intervention [31, 35]. On the other hand, to date ANNs have not been used for analyzing cytogenetic data or screening data in determining or predicting trisomies or any prenatal syndromes.

In this paper, instead of developing an automated method to classify chromosome classes used for determining and distinguishing between Turner, Patau, Klinefelter, Edwards and Down syndrome, we focus on developing an automatic scheme for classifying chromosomal trisomies using results from combined/double screening tests in the first trimester of pregnancy, ultrasonographic findings and maternal age. Validation of system output was done by karyotyping or known pregnancy outcome.

## Methods

### Sample collection

The database consisted of 2500 pregnant women data who received a routine antenatal care between January 2000 and December 2016. The first trimester combined test was offered routinely at 11 to 13.6 weeks of gestation, to measure the values of maternal serum levels of maternal serum pregnancy-associated plasma protein A (PAPP-A) and free beta human chorionic gonadotropin (β-hCG). All women underwent ultrasound examination also to acquire information on fetal nuchal translucency thickness and the presence or absence of the nasal bone.

Ultrasound verification was done on the General Electric 730 Volusion Exp, with convex probe 3.5–7 MgHz (multidimensional scanning). Additional parameter in the database was maternal age since the correlation between maternal age and presence of the prenatal syndrome has been proven [5]. After ultrasound examination, maternal blood was sampled using serum separator tubes (4 ml each) for the analysis. The serum was separated by centrifugation and stored at 4 °C until being tested. The blood samples were analyzed by using an automated Siemens Immulite 1000 system (Siemens Healthcare, Erlangen, Germany). Analysis of NT thickness, PAPPA-A and β-hCG, along with patient demographics was performed using the Prisca software (Siemens Healthcare, Erlangen, Germany). The pregnancy outcome was known for all the subjects included in the study known, either by amniocentesis or by childbirth. Those women with an elevated risk of (≥1 in 250) of carrying a fetus with trisomy 13, 18, or 21, and those women with advanced maternal age were offered counseling with the option for invasive diagnostic test. In cases when combined screening test estimated lower risk for trisomies, the outcome was further validated by childbirth.

Cytogenetic analysis on long-term cultured amniocytes was performed using standard manufacturer’s protocol (Amniomax, Invitrogen, Carlsbad, CA, USA). Metaphase chromosome spreads were prepared on a glass microscope slide in accordance with standard cytogenetic procedure and in accordance with the European Society of Human Genetics (ESHG) and European Cytogenetics Association (E.C.A) guidelines [36–38]. Chromosomes were aged and banded using G-Bands by pancreatin and Giemsa staining technique. To investigate the total number and structure of the chromosomes, twenty metaphase cells were visualized and analyzed by an experienced cytogenetics technologists using Zeiss microscope Axioskop2 plus (Zeiss, Jena, Germany) with the assistance of the MetaSystems imaging system. Results were reported in accordance with the International Standing Committee on Human Cytogenetic Nomenclature [11]. In the case of pathological karyogram findings, spouses were invited for genetic counseling at the Institute where multidisciplinary team (genetics, obstetrician and biologist) explained relevant medical facts regarding the findings. This diagnostic method was chosen due to availability since other invasive diagnostic testing methods such as chronic villi sampling (CVS) is not performed in Bosnia and Herzegovina, therefore it was not offered to any of our patients.

The dataset containing patient characteristics, test results, and pregnancy outcomes needed for development of the system was obtained from genetics laboratory at the Institute of Gynecology, Infertility and Perinatology “Mehmedbasic” in Sarajevo. In this dataset 1500 samples were of healthy subjects and 1000 samples were with diagnosis of aneuploidy.

Summary statistics of database is presented in Table 1. Out of 2500 observed samples, 52.5% were male and 47.5% were female samples. Minimum maternal age in this dataset is 16 years and the maximum age is 49 years. Mean maternal age of pregnant women who underwent amniocentesis is 31.5 years. Out of 2500 samples, 40% were with disease classification and 60% were of healthy subjects. Out of samples with confirmed diagnosis of prenatal syndrome most were of Klinefelter Syndrome (26.83%), followed with Down syndrome (24.31%), Edwards Syndrome (19.17%), Turner Syndrome (16.11%) and Patau Syndrome (13.58%). According to these samples, training dataset folds were created.

**Table 1:** Summary statistics for dataset

	Male		Female	
Gender	52.5%		47.5%	
	Min		Max	Mean
Maternal age	16		49	31.5
Syndrome classification data				
	Training dataset	Subsequent validationdataset	Total number of samples	Percentage of the overall dataset
Prenatal syndrome samples	800	200	1000	40%
Normal samples	1200	300	1500	60%
*Down Syndrome*	*292*	*73*	*243*	24.31%
*Edwards Syndrome*	*230*	*142*	*192*	19.17%
*Kleinfelter Syndrome*	*322*	*80*	*268*	26.83%
*Turner Syndrome*	*194*	*48*	*161*	16.11%
*Patau Syndrome*	*162*	*41*	*136*	13.58%

The Expert System based on Artificial Neural Network consists of 5 input parameters that are: maternal serum pregnancy-associated plasma protein A (PAPP-A), free beta human chorionic gonadotropin (β-hCG), mother’s age, fetal nuchal translucency thickness, the presence or absence of the nasal bone and one output parameter indicating that the tested subject has one of the prenatal syndromes or is healthy.

In designing neural networks for solving specific problem, factors such as neural network architecture, number of hidden neurons, training dataset distribution and training algorithm have significant impact on overall accuracy of developed system [39]. We investigated two different neural network architectures, feedforward and feedback, for various number of neurons in hidden layer, which are according to the application experts, sufficient to properly perform the classification [15, 40–42].

### Single hidden layer feedforward ANN with back-propagation

**Fig. 1:**
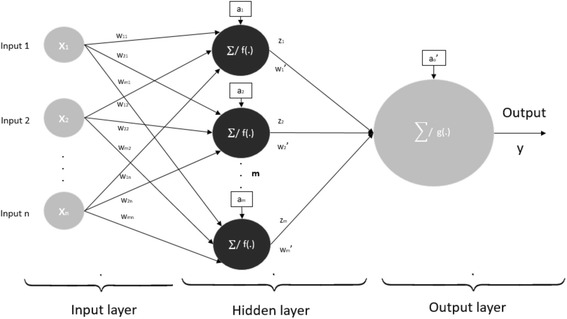
Architecture of a single layer feedforward neural network. n is number of inputs and m is number of neurons in hidden layer; f(·) and g(·) are transfer functions in hidden and output layer respectfully. Connections between neurons are represented with weight factors w; a is bias (internal neural network parameter); Σ indicates synapses – summation of signals from previous neurons

A feedforward neural network is probably the most common type of neural networks for classification and prediction [13]. As it can be seen from Fig. 1, network inputs are not affected with network output in any way. The output is result of modifiable synapses, represented as summations of signals from hidden and input layer.

In hidden layer, the inputs are linearly combined with vector of weights and biases (inner neural network parameter). The resulting linear combination of such input is then transformed to neuron output by transfer function. The output of single neuron in hidden layer is represented with following equation:

1 $$ {z}_i=f\left({a}_i+{\sum}_{k=1}^n{w}_{ik}{x}_k\right) $$

where *w*_*ik*_ is weight of *x*_*k*_ input to *z*_*i*_ output, and i is the number of neuron in hidden layer (1-m).

The output of ANN is formed as linear combination of hidden layer neuron outputs. These connections between hidden and output layer are also weighted. The output is calculated as:

2 $$ y=g\left({a_o}^{\hbox{'}}+{\sum}_{k=1}^m{w_i}^{\hbox{'}}{z}_i\right) $$

The transfer functions can be linear and nonlinear. To model non-linear relationship between inputs and outputs, like in cytogenetic analysis result and syndrome classification, non-linear transfer function such as logsig in hidden layer was used $\left( logsig(x)=\frac{1}{1+{e}^{-x}}\right)$. In output layer linear function (*purelin*(*x*) = *x*. Computationally, learning in this type of ANN architecture is regression process of adapting weights and biases until minimum error value is achieved. Simple iterative algorithm that can be used in training of this type of neural network is back-propagation algorithm. The output values are compared with the correct answer to compute the value of some predefined error-function. The weights and biases of neural network are corrected in each iteration. Most commonly used error function if mean square error (MSE). After training is completed, neural network parameters are held constant for subsequent analysis. This type of ANN can be used for modeling most problems while keeping simple architecture and low computational complexity. As a performance measure for trained neural network, absolute deviation from the actual output value can be used.

### Elman recurrent artificial neural network

**Fig. 2:**
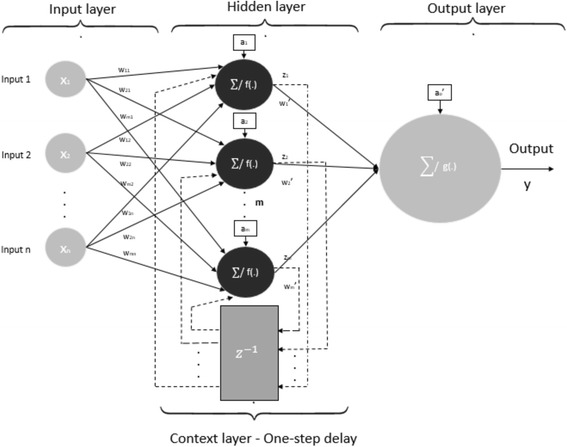
Elman neural network Architectures are context units that can be treated as the memory units. There are connections from the middle (hidden) layer to these context units fixed with a weight of one

Elman neural network has feedback (recurrent) architecture where connections between neurons form a directed cycle. The main difference between feedforward and feedback neural network is that feedback neural networks beside the input and output layer consist of recurrent layer which introduces one-step delay in hidden layer. This recurrent layer acts like memory in network architecture. This type of neural networks, because of this ability, is mostly used for recognition. Elman neural network is a three-layer network with vertically architecture shown in Fig. 2.

This neural network architecture also consists of input layer, hidden layer and output layer called the feedforward loop and context layer which makes back-forward loop with hidden layer which makes the neural networks sensitive to the history of input data. The hidden neuron outputs are calculated as follows:

3 $$ {z}_i=f\left({a}_i+{\sum}_{k=1}^n{w}_{ik}{x}_k(j)+{\sum}_{k=1}^m{w}_{ik}{z}_k\left(j-1\right)\right) $$

Where j stands for discrete time, *w*_*ik*_ is weight of *x*_*k*_ input to *z*_*i*_ output, and i is the number of neuron in hidden layer (1-m). The output is calculated as:

4 $$ y=g\left({a_o}^{\hbox{'}}+{\sum}_{k=1}^m{w_i}^{\hbox{'}}{z}_i\right) $$

The gradient descent (GD) algorithm is most commonly used for training of Elman’s neural networks. The error between the network output and the desired outputs minimized in the steepest descent [43]. At each iteration the input is propagated in a standard feed-forward architecture, and then a learning rule is applied. The back-forward loop results in the context units always maintaining a copy of the previous values of the hidden units, thus the network can maintain a sort of state, allowing it to perform such tasks as sequence-prediction that are beyond the power of a standard multilayer perceptron.

## Results

**Fig. 3:**
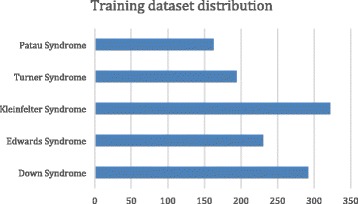
Training data set consisted of 1200 samples of normal subjects, 161 of Turner syndrome, 268 Klinefelter syndrome, 192 Edwards syndrome, 243 Down syndrome, 136 Patau Syndrome

Before developing neural network architecture, total database consisted of 2500 samples was divided into twodisjoint subsets, training and subsequent validation data. The training data consisted of 2000 samples (80% of overall database) and subsequent validation (testing of the system) was performed with the rest of the data as it can be seen from the Fig. 3. Out of 2000 samples 1200 were of healthy subjects and the rest were as follows: Klinefelter Syndrome 268 samples, Down syndrome 243 samples, Edwards Syndrome 192 samples, Turner Syndrome 161 samples and Patau Syndrome 136 samples, therefore in total 800 samples as presented in Table 1. This dataset distribution is common among researchers in the field when dealing with relatively small number of samples [44].

Due to database size and non-linearity of provided data for neural network training k-fold cross validation was used. The 2000 training samples were divided into 10 folds. This number of folds was chosen since the training dataset is comprised of 80% of database and according to experts in this division is sufficient [45]. In each iteration Levenberg – Marquardt algorithm (LMA) was used, which is common training algorithm in data classification [46]. The starting network weights were initialized with random values. At each training iteration train/test performance was evaluated as Mean Square Error between the predicted and actual values (MSE), where n is total number of samples:

5 $$ MSE=\frac{1}{n}{\sum}_{i=1}^n{\left({X}_{predicted}-{X}_{actual}\right)}^2 $$

The single performance estimation of neural network training process was generated by averaging results from k-folds. By using the k-fold cross validation the problem of over fitting was reduced. In case of over fitting the developed system has very good training performance but it’s predictive ability on unseen data is very poor. Developed neural network architectures were compared based on accuracy of classification of prenatal syndromes. To compare performance on neural network architectures, absolute error was used also. For validation purpose, absolute error is calculated as follows:

6 $$ {Error}_{absolute}={Output}_{target}-{ANN}_{output} $$

Dependency of classification accuracy in both architecture types to the number of neurons in hidden layer is examined. While number of input and output neurons is determined with data structure and process modeling, performance of training is dependent on complexity of neural network and number of neurons in hidden layer [30, 32]. Poorly defined number of neurons in hidden layer can cause over fitting problem, which leads to good training performance and very bad testing performance. There are various methods for choosing fixed number of neurons in hidden layer [47, 48], but there is no generally accepted one for determining the number of neurons in single hidden layer that would efficiently approximate any given function or process. Despite the new methods developed for this purpose, the most researchers use trial rule. This rule was used in this study also. Number of neurons in hidden layer is set to be 5, 10, 15, 17 and 20 and performance was calculated.

**Fig. 4:**
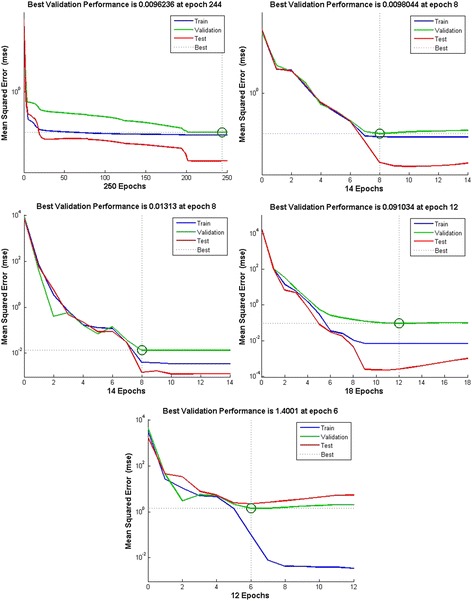
Training performance of Feedforward neural network with different numbers of hidden neurons

For each step detailed results are presented in Fig. 4 for feedforward neural network. The output performance is presented in Table 2. From the data presented in Fig. 4 and Table 3 it can be concluded that the best performance is achieved with 15 neurons for feedforward architecture.

**Table 2:** Feedforward model comparison based on different numbers of neurons in hidden layer

Number of hidden neurons	MSE calculation
5	0.0096
10	0.0075
15	0.0032
17	0.0309
20	0.0096

**Table 3:** Elman model comparison based on different numbers of neurons in hidden layer

Number of hidden neurons	MSE calculation
5	0.9535
10	0.7194
15	0.0856
17	0.3224
20	0.8710

**Fig. 5:**
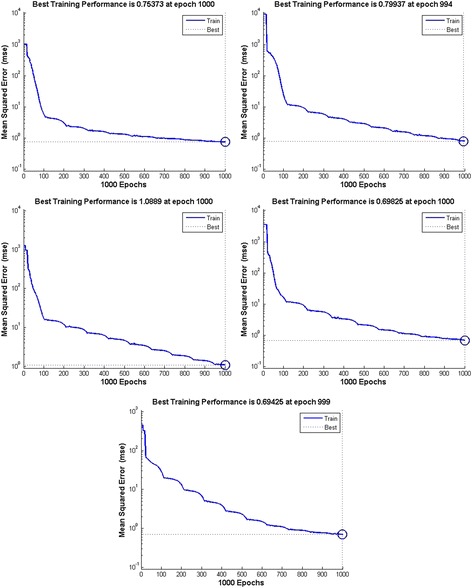
Training performance of Elman neural network with different numbers of hidden neurons

For each number of neurons, performance and absolute error of training dataset was calculated for feedback architecture also. Detailed results are presented in Fig. 5 and Table 3. From the data presented it can be concluded that the best performance is achieved with 17 neurons for feedback architecture.

**Fig. 6:**
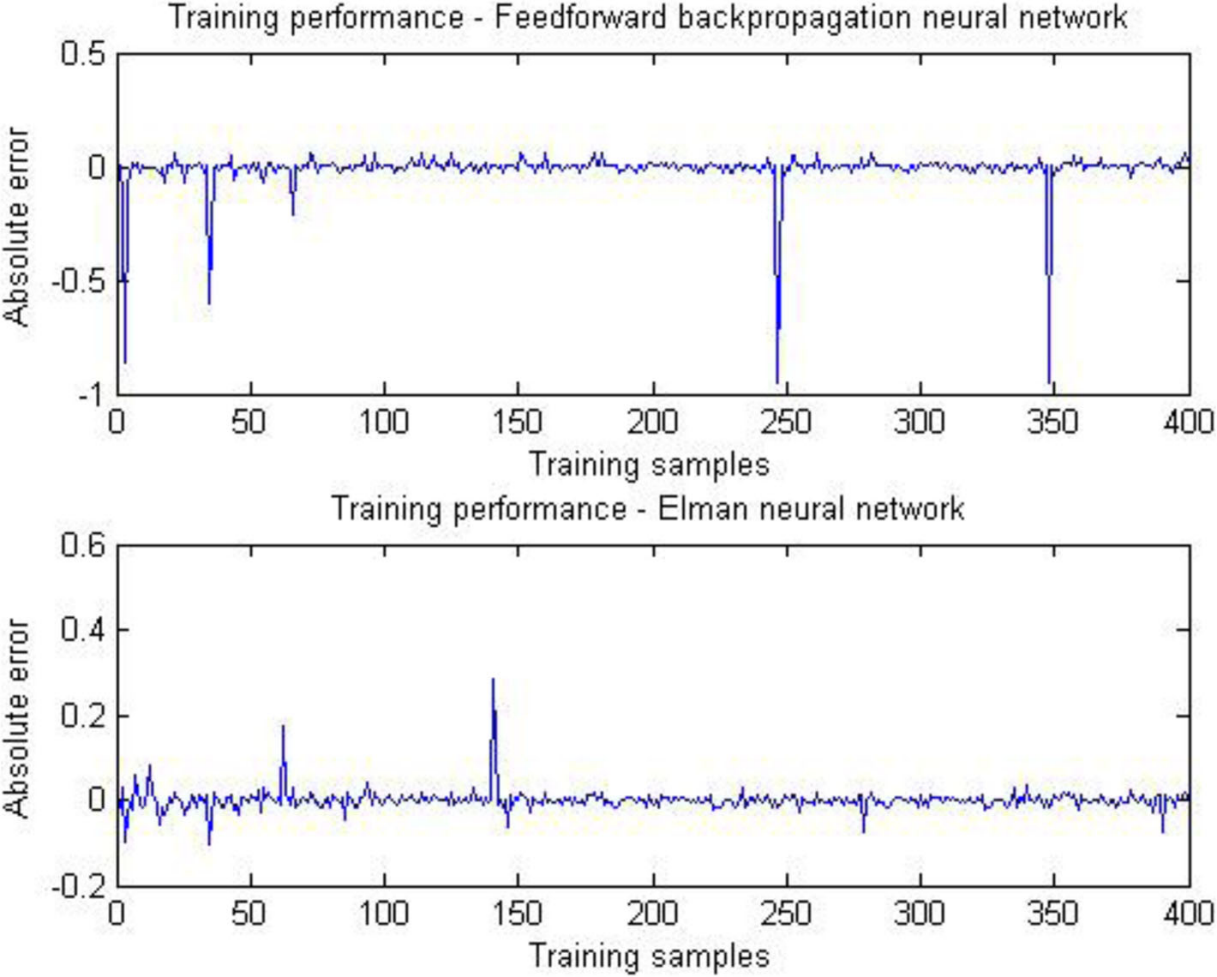
Training performance of Feedforward neural network (15 neurons) and Elman neural network (17 neurons)

Figure 6 shows the performance of architectures of neural networks chosen for further development of system for classification between prenatal syndromes. Those networks are feedforward neural network with 15 neurons and Elman neural network with 17 neurons. MSE for feedforward ANN was 15.6316 and for Elman’s ANN 1.5752.

Neural network subsequent validation performance for classification of five prenatal syndromes based on cytogenetic analysis and patient demographics is performed with 1000 samples out of which 1200 were healthy subjects, and 800 were subjects with disease with syndrome sample distribution as indicated in Table 1.

Based on data presented in Table 4, sensitivity of 92.00% was achieved for feedforward neural network architecture. The sensitivity of feedback neural network was higher and the value was 99.00%. Sensitivity was calculated based on following formula:

7 $$ Sensitivity=\frac{True\ postitive}{True\ postivie+ False\ negative} $$

**Table 4:** Feedforward neural network classification accuracy during subsequent validation

Feedforward Neural Network	Σ	Normal subject	Down Syndrome	Edwards Syndrome	Kleinfelter Syndrome	Turner Syndrome	Patau Syndrome
Normal subject	300	264	12	4	11	7	2
Down Syndrome	*51*	2	47	0	1	0	1
Edwards Syndrome	*42*	0	2	40	0	0	0
Kleinfelter Syndrome	*86*	1	0	2	81	1	1
Turner Syndrome	*15*	1	2	0	0	12	0
Patau Syndrome	*6*	2	0	0	0	0	4

While specificity was calculated based on:

8 $$ Specificity=\frac{True\ negative}{False\ positive+ True\ negative} $$

Detailed classification hits and misses of both neural networks are presented in Tables 4 and 5. Average accuracy of feedforward neural network was 89.6% and for feedback was 98.8% (Table 6).

**Table 5:** Elman neural network classification accuracy during subsequent validation

Elman Neural Network	Σ	Normal subject	Down Syndrome	Edwards Syndrome	Kleinfelter Syndrome	Turner Syndrome	Patau Syndrome
Normal subject	300	296	1	0	0	2	1
Down Syndrome	*51*	0	51	0	0	0	0
Edwards Syndrome	*42*	1	0	43	0	0	0
Kleinfelter Syndrome	*86*	0	0	0	85	0	1
Turner Syndrome	*15*	0	0	0	0	0	0
Patau Syndrome	*6*	0	1	0	1	0	4

**Table 6:** Subsequent validation performance results of developed neural networks

Total population	Σ	Feedforward ANN		Feedback ANN	
		Syndrome	Healthy	Syndrome	Healthy
Healthy	300	36	264	4	296
Syndrome	200	184	16	198	2
Sensitivity [%]		92.00%		99.00%	
Specificity [%]		88.00%		98.67%	
Average accuracy		89.6%		98.8%	

## Discussion

To design an expert diagnostic system based on neural networks for classification of prenatal aneuploidy syndromes, we presented a novel method based on the results of combined/double test in the first trimester of pregnancy, ultrasonographic findings and maternal levels of free β-hCG and PAPP-A. The Expert System technique proposed in this paper potentially opens new avenues for the development of inexpensive, yet effective, prenatal aneuploidy screening tests. The simplicity and accuracy of this method make it a good candidate for clinical implementation as standard software for screening procedure.

There has been significant increase in usage of artificial neural networks in medicine [49], and specifically, in pediatrics. There are several studies based on determining one out of five prenatal syndromes analyzed in this study. Wojtowicz et al. [50] in their study use phenotypic features in diagnosing Down syndrome, postnatal, based on features versus genotype data and patient demographics that were done prenatal. In other study Wojtowicz et al. [51] discuss a new system that is used to solve the problem of the recognition of the dermatoglyphic pattern and the understanding of the classification process of the symptoms of Down syndrome. This system is based on the combination of text knowledge found in the scientific literature describing Down syndrome with the knowledge obtained from the analysis of dermatoglyphic indices characteristic of Down syndrome with the use of digital pattern recognition techniques. Instead of numerical values used for classification of prenatal syndromes in this study Wojtowicz et al. [51] use pattern recognition algorithms for determining the Down syndrome.

Also, for postnatal determination of Turner syndrome Naïve Bayesian neural network has been proposed in study by Pereira et al. [52]. The syndrome classification was based on nine input parameters, phenotype attributes. The problem encountered in building the Bayesian network and the forming of an expert probabilistic system was to obtain knowledge, i.e. database of useful experiment results.

A large number of different neural network structures have been constructed, trained and tested to a large database of pregnant women characteristics, aiming at generating aclassifier-predictor for the presence of chromosomal abnormalities. Soleimani et al. [53] in their study used 3-layer artificial neural network with back propagation learning algorithm to predict development disorders based only on perinatal data. True prediction of developmental disorder, obtained in this study was 83.1%. In our study, both feedforward and feedback neural network architecture used Levenberg – Marquadat training algorithm and exhibit higher accuracy than this multilayer neural network explored in previously mentioned study.

Nicolaides et al. [54] in their study used artificial neural network for non-invasive chromosomal abnormality screening. The best results were obtained when using a multi-layer neural structure having an input, an output and three hidden layers. The percentage of abnormal cases correctly predicted was 85.1%. In the same study, Turner syndrome was predicted with 42.9%. In our study, classification of Turner syndrome was higher when Elman architecture was used and lower than mentioned one when feedforward architecture was used. Also mentioned researchers incorporated “genetics” results to compare or to show “false negative and false positive” results.

Other analysis application of neural networks in prenatal diagnostics relay on using these systems for chromosome identification. For instance, Wang et al. [40] built multi-feature ANN metaphase chromosome classifier based on 150 metaphase chromosome cell images (2300 individual chromosomes) from both normal and abnormal peripheral blood and amniotic fluid samples. Similar to our ANN design, their ANNs had feed-forward structure with three layers composed of: input, hidden and output neurons.

In the future work, the aim of the researchers is to develop Graphical User Interface for implemented Expert System, as it is practice in the other similar studies [55], so physicians will be able to use it in friendlier environment.

## Conclusions

A chromosome disorder is caused by an alteration in the number or genetic structure of chromosomes. A trisomy is a chromosomal disorder characterized by an additional chromosome, meaning that the affected person has 47 chromosomes instead of 46. The most common forms of trisomy are: Down syndrome, Edwards syndrome, Patau syndrome and Klinefelter. Children affected by trisomy usually have a range of birth defects, including delayed development and intellectual disabilities.

Cytogenetic and advanced molecular analyses are convincing prenatal diagnostic investigation along with clinical suspicions and biochemical screening tests. Microscope analysis and computer imaging are the most common methods used for prenatal diagnostics. However, visual karyotyping using microscopic images is time-consuming and labor intensive, which can reduce the diagnostic efficiency and accuracy. Hence, using computer-based expert diagnostic systems can significantly contribute to the evaluation of prenatal diagnostic screening test results and can be an adjunct to routine chromosomal analysis.

Aneuploidies involving 13, 18, 21 and sex (X and Y) chromosomes account for the majority of all chromosome abnormalities in live-born infants. The risk of chromosomal abnormality in the fetus increases with increasing maternal age. Rapid diagnosis of fetal chromosome anomalies may facilitate clinical decision-making, especially when a fetal abnormality is detected late in pregnancy.

In most cases, the loss of a whole chromosome is incompatible with life and will result in early miscarriage or stillbirth. If the extra genetic material is an entire chromosome, the effect is largely lethal.

In this study, we used two neural network architectures to classify between five prenatal syndromes based on the results of maternal serum screening tests, ultrasonographic findings and patient demographic. The aim of this work was to examine efficiency of different neural network architectures for this task. This study has proven that relatively simple neural network architecture, such as feedforward, can have high classification accuracy. Because of the non-linear input-output relationship better accuracy of classification can be achieved with recursive neural network architecture, such as Elman architecture. To our knowledge, this is the first application of ANN developed for classification offirst trimester maternal serum screening samples. Nonetheless, we expect that this ANN system can be expanded to other syndromes and other sample types. This supports the potential use for the fast and reliable detection of genetic disorders and fetal well-being.

There are several limitations in thisstudy. Forinstance, the use of a single health center and single equipment during the study. The second important limitation of the study is the limited number of “abnormal” samples. Nevertheless, the analysis program’s performance is limited to input information and knowledge and medical expert cannot get more than he or she has donated the system.

The impact of developed system is that it enables classification of prenatal syndromes based on input parameters that can be acquired with relatively non-invasive and low cost methods. Developed system comprises of expert knowledge in this field since all diagnosis were previously revised by field professional. By enlarging the database with various datasets regarding demographics system accuracy can be improved and the usage of the system expanded to other regions. This system can be used instead of commercial prenatal syndrome screening software’s especially in low-income countries where healthcare systems are dealing with rising costs of diagnostic and disease treatment.

## Abbreviations

aCGH: Array comparative genomic hybridization
ANN: Artificial Neural Network
A-PAPP-A: Pregnancy-associated plasma protein
CVS: Chorionic villus sampling
FISH: Fluorescence in situ hybridization
GD: Gradient descent
LMA: Levenberg-Marquardt algorithm
MSE: Mean Square Error
NGS: Next-generation sequencing
QF-PCR: Quantitative fluorescence polymerase chain reaction
β-hCG: beta human chorionic gonadotropin
